# Quality of life in Croatian Homeland war (1991-1995) veterans who suffer from post-traumatic stress disorder and chronic pain

**DOI:** 10.1186/1477-7525-9-56

**Published:** 2011-07-29

**Authors:** Marijana Braš, Vibor Milunović, Maja Boban, Lovorka Brajković, Vanesa Benković, Veljko Đorđević, Ozren Polašek

**Affiliations:** 1 Centre for Palliative Medicine, Medical Ethics and Communication Skills, Medical School, University of Zagreb, Zagreb, Croatia; 2 Clinical Hospital Merkur, Zagreb, Croatia; 3 Care of Children and Youth Zagreb, Zagreb, Croatia; 4 Croatian Society for Pharmacoeconomics and Health Economics, Zagreb, Croatia; 5 Department of Public Health, Medical School, University of Split, Split, Croatia

## Abstract

**Background:**

The aim of this study was to investigate the quality of life in Croatian homeland war veterans who suffer from post-traumatic stress disorder and chronic low back pain (LBP).

**Methods:**

A total of 369 participants were included, classified in four study groups: those with post-traumatic stress disorder (PTSD; N = 59), those with both PTSD and lower back pain (PTSD+LBP; N = 80), those with isolated LBP (N = 95) and controls (N = 135). WHOQOL-BREF survey was used in the estimation of quality of life. The data were analysed using statistical methods and hierarchical clustering.

**Results:**

The results indicated a general pattern of lowering quality of life in participants with both psychological (PTSD) and physical (LBP) burden. The average overall quality of life was 2.82 ± 1.14 for the PTSD+LBP group, 3.29 ± 1.28 for the PTSD group, 4.04 ± 1.25 for the LBP group and 4.48 ± 0.80 for the controls (notably, all the pair-wise comparisons were significantly different at the level of P < 0.001, except for the pair LBP-controls, which was insignificant). This result indicated that quality of life was reduced for 9.9% in patients with LBP, 26.6% in patients with PTSD and 37.1% in PTSD+LBP, suggesting strong synergistic effect of PTSD and LBP. The analysis also identified several clusters of participants with different pattern of quality of life related outcomes, reflecting the complex nature of this indicator.

**Conclusions:**

The results of this study reiterate strong impact of PTSD on quality of life, which is additionally reduced if the patient also suffers from LBP. PTSD remains a substantial problem in Croatia, nearly two decades after the beginning of the 1991-1996 Homeland war.

## Background

Posttraumatic stress disorder (PTSD) is an extreme response to a traumatic event characterized with persistent re-experiencing of the trauma through recurrent and intrusive recollections or dreams, persistent avoidance of stimuli associated with the trauma, numbing of general responsiveness and persistent symptoms of increased arousal [1]. The net result of all these changes includes a wide range of dysfunctions and personal maladjustments [2-4], as well as a reduction of the overall quality of life [5]. PTSD most frequently occurs among combat veterans who experienced wartime-related psychological traumas [6-8].

However, it seems that a simple exposure to a stressful event is not crucial for the disease development, although the amount of stress is proportional to the chances of developing the disease. A study on Vietnam war veterans has shown that 26% of those involved in severe combat have developed PTSD, 17% of those involved in moderate-level combat and only 7% of those who were not directly involved in combat have developed PTSD [9]. Studies on Croatian Homeland war veterans have shown that 16% of veterans suffered from fully developed PTSD and 26% of them had sub-clinical manifestations [10].

Despite numerous treatment approaches and schemes, subsets of PTSD patients develop a chronic unremitting disorder disease with a lifelong course [11]. One of the main disadvantages of this disease is related to the existence of chronic pain, which may be affecting as much as 80% of the PTSD population [12,13]. Chronic pain can also be considered to be a stressor that exceeds routine coping capacities, which in turn may lead to disability and reduction in the overall quality of life [14-17]. A number of studies have investigated the negative effects of chronic pain or PTSD on the quality of life so far [18-24], but studies that investigated the relationship of chronic pain in combat-induced PTSD and quality of life based on the standardized survey approach in a veteran population are scarce.

Therefore, the aim of this study was to investigate the differences in quality of life within four groups of participants: war veterans with established PTSD, war veterans with both PTSD and lower-back chronic pain, war veterans who only suffered from lower-back pain without PTSD and lastly controls, who were without any of these conditions.

## Methods

### Participants

A total of 536 participants were initially contacted in this study. They were selected to represent a population of Croatian Homeland war veterans, aged 35-54 years, who were exposed to direct combat conditions for at least three consecutive months. The participants were included in the present study by the means of consecutive enrolment at the Clinic for Psychological Medicine, Clinical Hospital Centre in Zagreb and Clinic for psychiatry, Clinical Hospital Centre in Osijek in the period of 2008-2009. The sample was additionally supplemented with smaller number (N = 40) of participants who were involved through the means of direct contact with some of the veterans' nongovernmental organizations, representing groups of veteran population that maintain contacts and share their issues. The sample structure was thus aiming at provision of the wide range of PTSD sufferers, in order to provide a good mix of those with mild and those with more severe clinical manifestations of the disease. We aimed to create four study groups of approximately same size of 100 participants:

1. war veterans suffering from chronic PTSD and lower-back pain (LBP)

2. war veterans suffering from chronic PTSD only

3. war veterans suffering from chronic LBP only

4. war veterans who were at the time of study showing none of these disorders (healthy controls)

In order to classify the participants into these four groups, we undertook a number of diagnostic procedures. Firstly, all participants were interviewed by an experienced psychiatrist at the Clinic for Psychological Medicine, University Hospital Centre, Zagreb to assess the presence of PTSD according to DSM-IV-TR criterion. The diagnoses were established at different times during or after the war and were re-evaluated in regular prior to this study. Final evaluation of PTSD diagnosis was made at the time of the study. Participants with positive anamnesis of head and spinal injury, acute psychosis, alcohol or illegal substance abuse or those who were diagnosed with any form of the psycho-organic syndrome were excluded from the study. After establishing a PTSD status, we proceeded to classify them according to the lower-back pain status. Initial criterion was the presence of LBP with a minimal duration of 12 months. Participants who reported suffering from LBP were then directed to a specialist surgeon at the Clinic for Traumatology, Zagreb for further clinical and radiographic testing by means of magnetic resonance imaging. In order to exclude participants with herniation and sciatica or detectable organic causes that were not in line with the patient's age, we used magnetic resonance imaging by a 1.5 T Magneton Symphony (Siemens Medical System). T1 weighted scans were used to assess anatomic relations and T2 weighted scans were used to assess pathologic change of signal. Repetition time totaled 510-810 ms; echo time 14-17 ms. The slice thickness was 2-3 mm. Field of view was 120-180 mm with matrix of 512 × 256. In order to suppress possible bias, radiologists were unaware of the patient's conditions and were asked to report the presence of lumbar disc degeneration, protrusion, herniation and spinal stenosis. By doing this, we were able to classify all participants into positive or negative PTSD and LBP group. Participants who had no indication of PTSD nor reported LBP were considered to be healthy controls.

Overall response rate was 81%, i.e. participants completing all diagnostic procedures, with 65 of participants being excluded according to the exclusion criteria listed above. The final sample for this study consisted of a total number of 369 participants, assigned to the four study groups: 80 participants who were considered to have both PTSD and LBP, 59 of those who had isolated PTSD, 95 with LBP and 135 controls. Each participant signed an informed consent and the study was conducted by ethical principles set by WMA Declaration of Helsinki. The study was approved by the Ethical Board of the Clinic for Traumatology in Zagreb.

### Questionnaires

A general questionnaire was developed to assess basic demographics, LBP status and psychiatric data. Items assessing LBP included various risk factors such as weight, height, body mass index, vocational activity, various LBP descriptors such as duration of symptoms, intensity and potential use of analgesic medications. Items analyzing PTSD included duration PTSD, onset of symptoms, other co-morbid psychiatric disorders, psychotropic medication, war exposure, short description of traumatic events.

All participants were also given World Health Organization Quality Of Life-BREF questionnaire (WHOQOL-BREF)[18], including set of general questions, regarding age, sex, socio-economic status, other co-morbid psychiatric disorders and physical disorders. The WHOQOL-BREF assessment is a self-reported questionnaire that contains 26 items, and each item represents 1 facet. The facets are defined as those aspects of life that are considered to have contributed to a person's QOL. Among those 26 items, 24 of them make up the 4 dimensions of physical health (7 items), psychological health (6 items), social relationships (3 items), and environment (8 items), whereas the other 2 items measure overall QOL and general health. Respondents rated the intensity, frequency, or evaluation of the selected attributes of QOL during the previous 2 weeks on a 5-point Likert-response scale.

### Statistical analysis

For statistical analysis, the WHOQOL-BREF assessment was first summarized to a 4-dimension construct (physical health, psychologic health, social relationships, environment) according to the guidelines for the WHOQOL-BREF. All dimension scores were calculated by taking the mean score for all items included in each dimension and multiplying by a factor of 4, where higher score indicating better QOL. The data were analysed using variance analysis, with either LSD or Dunnet T3 used as post-hoc tests (depending on the sample variance homogeneity, which was estimated using Levene's test). Fisher's exact test was used for categorical data analysis, due to small number of participants in some contingency tables.

In order to show the overall pattern of quality of life across study groups, we used a comprehensive approach involving hierarchical clustering and factor analysis. Firstly, we performed a hierarchical clustering which included all 26 questions from the WHOQOL-BREF questionnaire. The method was based on squared Euclidian distance. The number of clusters was defined in an ascending order, ranging from 4 to 10, in order to find the most informative cluster. After the clustering was completed, we recorded the predicted clustering group membership and compared it to the original study groups. Additionally, in order to show how these clusters were made, we reported mean value and standard deviations for the first questionnaire question for each predicted cluster group. We also performed a factor analysis of the same set of questions in order to obtain dimensionality reduction. Three dimensions were extracted using varimax rotation, which explained 63.7% of entire variance. Lastly, we made multinomial logistic regression models, which were predicting the differences between the three analysed groups and controls. Statistical analysis was performed using the SPSS, version 16.0, with significance set at P < 0.05.

## Results

A total of 369 participants were included in this study, falling into four study groups (Table 1). The initial analysis suggested that these four groups were different in most basic characteristics, including age, employment and marital status, amount of smoking and self-reported physical activity, while we did not detect a significant difference in the educational structure of sub-samples (Table 1).

**Table 1:** Basic comparison of the four investigated groups

		PTSD + LBP (N = 80)	PTSD (N = 59)	LBP (N = 95)	Controls (N = 135)	P
Age; mean ± standard deviation		45.20 ± 5.05	43.32 ± 5.45	48.60 ± 4.98	41.78 ± 5.40	< 0.001
Education; n (%)	Primary school	10 (14.5)	4 (12.1)	10 (14.9)	12 (12.9)	0.499
	Secondary school	49 (71.0)	25 (75.8)	39 (56.7)	65 (69.9)	
	College degree	7 (10.1)	2 (6.1)	9 (13.4)	7 (7.5)	
	University degree	3 (4.3)	2 (6.1)	10 (14.9)	9 (9.7)	
Employment status; n (%)	Employed	14 (20.6)	5 (12.5)	48 (51.10)	98 (73.7)	< 0.001
	Pensioned	46 (67.6)	33 (82.5)	35 (37.2)	7 (5.30)	
	Unemployed	8 (11.8)	2 (5.0)	11 (11.7)	28 (21.1)	
Marital status; n (%)	Married	56 (80.0)	30 (73.2)	75 (79.8)	67 (50.8)	< 0.001
	Single	6 (8.6)	6 (14.6)	9 (9.6)	61 (46.2)	
	Living together with a partner	3 (4.3)	2 (4.9)	4 (4.3)	2 (1.5)	
	Divorced	4 (5.7)	3 (7.3)	3 (3.2)	1 (0.8)	
	Widoved	1 (1.4)	0 (0)	3 (3.21)	1 (0.8)	
Smoking; n (%)	Don't smoke	43 (61.4)	21 (52.5)	69 (73.4)	84 (62.7)	0.015
	Smoke, less than 20 cigarettes per day	11 (15.7)	10 (25.0)	13 (13.8)	37 (27.6)	
	Smoke, more than 20 cigarettes per day	16 (22.9)	9 (22.5)	12 (12.8)	13 (9.7)	
Self-reported physical activity; n (%)	No physical activity	21 (29.4)	7 (17.9)	18 (19.8)	11 (9.2)	< 0.001
	Occasionally	35 (51.5)	18 (46.2)	44 (48.4)	45 (37.5)	
	Once a week	2 (2.9)	5 (12.8)	15 (16.5)	23 (19.23)	
	Daily	11 (16.2)	9 (23.11)	14 (15.4)	41 (34.2)	

The analysis of the dimensions of quality of life survey in the four investigated groups indicated the presence of strong differences in the four distinctive WHOQOL-BREF dimensions, except for the difference in PTSD vs. PTSD and LBP group (Table 2).

**Table 2:** The comparison of quality of life among study groups using analysis of variance and post-hoc tests

QOL dimension/status	Groups	N	Mean ± SD	F; P	*Post-hoc *differences*
Physical	PTSD + LBP (I)	79	75.44 ± 11.33		
	PTSD (II)	56	78.43 ± 11.54	49.18;	I-III, I-IV, II-III,
	LBP (III)	84	87.43 ± 13.84	< 0.001	II-IV, III-IV
	Controls (IV)	134	94.42 ± 11.65		
	Total	353	85.97 ± 14.40		
Psychological	PTSD + LBP (I)	76	63.74 ± 14.60		
	PTSD (II)	58	67.45 ± 15.92	79.05;	I-III, I-IV, II-III,
	LBP (III)	90	80.27 ± 14.59	< 0.001	II-IV, III-IV
	Controls (IV)	132	90.67 ± 10.76		
	Total	356	78.51 ± 17.44		
Social	PTSD + LBP (I)	80	33.40 ± 8.89		
	PTSD (II)	58	35.93 ± 9.98	70.19;	I-III, I-IV, II-III,
	LBP (III)	91	41.58 ± 8.78	< 0.001	II-IV, III-IV
	Controls (IV)	134	49.22 ± 7.13		
	Total	363	41.70 ± 10.6		
Enviromental	PTSD + LBP (I)	79	92.81 ± 20.78		
	PTSD (II)	58	100.76 ± 19.79	66.27;	I-III, I-IV, II-IV,
	LBP (III)	88	108.36 ± 17.71	< 0.001	III-IV
	Controls (IV)	130	126.06 ± 14.27		
	Total	355	110.14 ± 22.02		
Satisfaction with personal health status	PTSD + LBP (I)	80	1.84 ± 0.74		
	PTSD (II)	59	2.36 ± 0.85	127.48;	I-II, I-III, I-IV, II-IV,
	LBP (III)	95	2.70 ± 0.98	< 0.001	III-IV
	Controls (IV)	135	4.03 ± 0.85		
	Total	369	2.94 ± 1.23		
Overall self-reported quality of life	PTSD + LBP (I)	73	2.82 ± 1.14		
	PTSD (II)	49	3.29 ± 1.28	24.04;	I-II, I-III, I-IV, II-III,
	LBP (III)	75	4.04 ± 1.25	< 0.001	II-IV
	Controls (IV)	42	4.48 ± 0.80		
	Total	239	3.59 ± 1.31		

*Group-by-group comparisons that were significant at the level of P < 0.001 performed using LSD (homogenous variance; used for physical and overall quality of life) or Dunnet T3 (unhomogenous variance; all other questions). The significance was set at P < 0.001 in post-hoc test in order to reduce the increased chances of false positive results.

We also did not detect a significant difference in comparison of PTSD vs. LBP for environmental dimension (Table 2). Furthermore, when the question on the overall quality of life was analysed, the results indicated that the group of patients who had PTSD and LBP had much worse quality of life than those with PTSD only, suggesting a synergistic effect of physical disorder in the form of LBP and psychological disruption in the form of PTSD (Table 2). It should also be noted that we did not detect a significant difference in this question between controls and patients with LBP only (Table 2).

**Figure 1:**
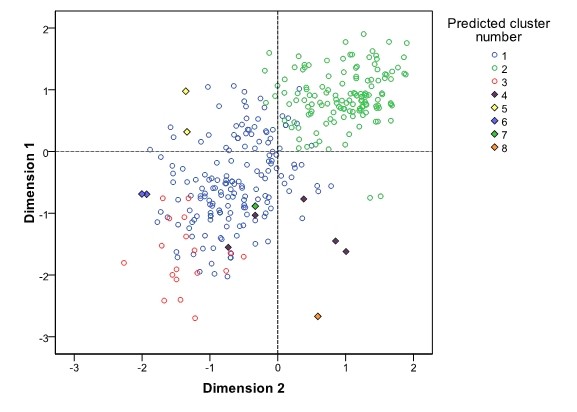
**Scatter plot showing the first two dimensions from factor analysis and representing the two-dimensional distribution of predicted cluster memberships according to all 26 items in the WHOQOL-BREF questionnaire**. Clusters of size with five or less participants are represented by fully coloured rhomboid shapes.

In order to examine the quality of life among the participants from four study groups, we also performed a hierarchical clustering analysis, aiming to show the distances among all included participants. The comparison of the original study group membership with the cluster-based predicted membership indicated interesting pattern of cross-correlations between these two variables (Table 3). Two large clusters were obtained, two small and four clusters with either one or two participants in them. Comparison of these clusters indicated that the second predicted cluster had the highest mean quality of life, with predominant membership from controls; predicted cluster number 1 had worse mean quality of life and was predominantly receiving membership from the PTSD+LBP and PTSD group, while cluster number 3 had most contribution from the PTSD+LBP group and had the lowest mean quality of life (Figure 1). The multivariate analysis repeated most of the results from the previous analytic steps, including differences in some of the basic descriptive characteristis, but also extending across dimensions of the quality of life (Table 4). A general pattern indicated the greatest deviation from the controls in terms of worse outcomes in the overall quality of life and some dimensions in PTSD+LBP group, while isolated LBP group seemed to differ the least strongly from control group (Table 4).

**Table 3:** Cross-correlation of the study groups with predicted cluster membership based on the hierarchical clustering; summary statistics is presented for clusters of equal or greater size than five participants

Predicted cluster number	Study group				Total	Mean ± SD, QOL1	Cluster distribution P*
	PTSD+LBP	PTSD	LBP	Controls			
1	62 (77.5)	46 (78.0)	56 (58.9)	17 (12.6)	181	2.93 ± 0.80	< 0.001
2	1 (1.3)	6 (10.2)	37 (38.9)	115 (85.2)	159	4.11 ± 0.61	< 0.001
3	14 (17.5)	2 (3.4)	1 (1.1)	1 (0.7)	18	1.44 ± 0.62	< 0.001
4	1 (1.3)	3 (5.1)	1 (1.1)	0 (0)	5	2.60 ± 0.89	0.004
5	1 (1.3)	1 (1.7)	0 (0)	0 (0)	2	-	-
6	0 (0)	0 (0)	0 (0)	2 (1.5)	2	-	-
7	1 (1.3)	0 (0)	0 (0)	0 (0)	1	-	-
8	0 (0)	1 (1.7)	0 (0)	0 (0)	1	-	-
Total	80	59	95	135	369	-	-

*Due to small number of participants in some groups Fisher's exact test was used

**Table 4:** Results of the multinomial logistic regression analysis where each of the three study groups were contrasted to controls in order to show their specific deviations from the control group

	PTSD+LBP		PTSD		LBP	
	P	OR [95% CI]	P	OR [95% CI]	P	OR [95% CI]
Age	0.164	1.07 [0.97-1.18]	0.818	1.01 [0.91-1.12]	< 0.001	1.26 [1.14-1.39]
Employment status	0.015	2.57 [1.20-5.52]	0.001	3.43 [1.61-7.32]	0.484	1.29 [0.63-2.65]
Marital status	< 0.001	0.32 [0.17-0.60]	0.005	0.40 [0.21-0.76]	0.057	0.59 [0.35-1.01]
Smoking	0.152	1.62 [0.84-3.15]	0.017	2.21 [1.15-4.24]	0.724	0.88 [0.45-1.75]
Education	0.689	1.16 [0.56-2.41]	0.351	1.42 [0.68-2.98]	0.298	1.42 [0.73-2.77]
Pysical activity	< 0.001	0.39 [0.24-0.64]	0.060	0.62 [0.37-1.04]	0.001	0.46 [0.28-0.73]
Quality of life dimensions						
Physical	0.916	1.00 [0.95-1.06]	0.976	1.00 [0.95-1.06]	0.207	1.03 [0.98-1.09]
Psychological	0.012	0.94 [0.89-0.99]	0.004	0.93 [0.88-0.98]	0.209	0.97 [0.92-1.02]
Social	0.197	0.95 [0.88-1.03]	0.643	0.98 [0.91-1.06]	0.858	0.99 [0.92-1.07]
Environmental	0.006	0.95 [0.91-0.98]	0.115	0.97 [0.93-1.01]	0.049	0.96 [0.92-1.00]
Overall quality of life	< 0.001	0.15 [0.05-0.39]	0.010	0.14 [0.03-0.62]	0.058	0.45 [0.18-1.03]

## Discussion

The results of this study show a general pattern of diminishing quality of life with the increasing psychical and physical burden in an individual. The control group of health participants had the best indices of quality of life, which seemed to be the least affected by the presence of physical pain only (LBP); quality of life diminished more in the presence of psychological disruption (PTSD), while it diminished even more in some dimensions in patients who suffered from both psychical and physical problems (PTSD and LBP). However, it should also be noted that we did not detect significant difference between patients who had isolated PTSD from those who had PTSD and LBP in all four dimensions of quality of life estimation, thus suggesting the fine interplay and sometimes unclear boundary between these two groups of patients. This type of synergistic action of different stressors was described before, suggesting a complex interplay of factors that affect the quality of life in patients with PTSD [25].

Worsening of the quality of life in patients suffering from PTSD has been reported in previous studies in the comparable effect size [26-28]. Even the results from the previous study on Croatian veterans indicated similar result, which was especially strongly expressed in the social dimension, showing that the effect of emotional numbing could have devastating consequences for these patients [29]. The results of this study confirmed such result, with the social dimension being the most strongly affected among four principal WHOQOL-BREF dimensions (Table 2). This result fits in well with the results from a large meta-analysis which indicated that inadequate social support after the traumatic event may act as a moderate predictor for the occurrence of PTSD [30]. Impaired quality of life in patients with PTSD was also confirmed in situations when PTSD originated from reasons other than war, showing similar effect size and pattern of changes [31-33]. The consistency of these results indicates that regardless on the mechanism that led to the PTSD diagnosis, the reduction of the quality of life was evident in these patients. The results of this study extend the previous ones, in a sense that they are showing even further reduction in situations when a physical component is present, suggesting a multi-dimensional nature of the determinants of quality of life in these patients.

The use of more advanced statistical analytic methods indicated the existence of several cluster of quality of life, suggesting a presence of several patterns. Majority of those who reported better quality of life were from the control group and LBP group, while majority of those who reported worse QOL were from the PTSD and PTSD with LBP group. Nevertheless, there were some exceptions, suggesting that the measurement and estimation of QOL and its association with these diagnoses not as clear as one might suggest. This, coupled with the presence of several distinctive clusters made up of several individuals suggests that there might be other mechanisms that determine or modulate this association, and that the diagnosis is just one part of the QOL determination. Such modifying effects could be residing in a number of possible effects, including personal coping capabilities, personal characteristics, societal support or other. It is those outliers that are actually very interesting in the broader perspective, especially knowing that the therapeutic opportunities for these conditions (PTSD) are often ineffective and when they do produce effect they must be given in a form of a lifelong therapy. This is of special interest in countries that underwent substantial causal events such as Croatia in the post-war period. Nearly two decades after the war has started, Croatia is struggling with the consequences of the war. The official data suggests that the total cost of war was estimated at $37.4 billion USD, up to 20,000 persons have been reported killed or missing, and more than 30,000 people have been disabled as a result of the war [34]. The societal impact of this is enormous, despite the fact that the real number of people suffering from PTSD and other disorders is very difficult to estimate, due to difficulties in diagnosing mild cases and the fact that a person can develop PTSD years after the exposure to traumatic event [35].

The other implication of this study lies in the possible identification of specific PTSD subpopulation (cluster 3) suffering from chronic LBP associated with significant deterioration in QOL as seen in Figure 1. Although this association between chronic pain syndromes and PTSD has been established by some studies, most recently National Comorbidity Survey-Replication with PTSD having a high likelihood for chronic pain disorder (OR = 5.4, 95% CI [3.6-7.9]), this area is underdeveloped with scarce data about possible neural correlates and treatment of chronic pain in PTSD [36-38]. Furthermore, the recent findings by Spoont et al. in a large cohort of veterans with newly diagnosed PTSD from VA facilities and primary practice suggesting that only a minority of these patients receive adequate treatment indicating that PTSD is still insufficiently treated [39]. Our results present the deterioration of QOL as an possible indicator of inadequate PTSD treatment. Chronic PTSD with other comorbid pain disorder thus represents a challenge to a proper clinical management, warranting further research.

The limitations of this study include possible difficulties in establishing PTSD, especially since most of the diagnoses were set long time ago, in some cases even during the war. However, this result also suggests the chronic nature of the disease, reflected through the inability of these patients to obtain satisfactory quality of life even two decades after the traumatic experience. There is also a possible bias in this, as we could have included only those patients who have very resistant from of PTSD that lasts for almost two decades, thus perhaps even overestimating the results and the difference in quality of life. The WHOQOL-BREF survey contains only three items related to social dimension of the quality of life, thus suggesting that the results obtained here could be to a certain level imprecise. This raises the question of the appropriate QOL tool for future studies on PTSD, which should focus on the social dimension more. Another limitation of this study is rather small sample size, in a sense that some finer scale and more subtle differences could not have been uncovered and that larger sample sizes might be more appropriate to detect these differences. Furthermore, PTSD also bears potential to cause psychological changes of an individual thus causing further difficulties is estimation of true quality of life, and is also prone to various levels of other possible known and unknown confounders that could affect the results.

## Conclusions

The results of this study suggest a synergistic effect of PTSD and low back pain on reduction of the quality of life in patients suffering from both diagnoses. These results are in line with the general pattern of expectation, where increased psychological and physical load in an individual leads to impaired quality of life. The degree of the change seems to be dependant on a number of factors, but patients who suffer from both PTSD and LBP show even worse quality of life that those with PTSD only, despite rather low effect size reported in this study. PTSD remains a substantial problem in Croatian health care, with large number of reported cases and high overall burden for both health system and society in total.

## Competing interests

The authors declare that they have no competing interests.

## Authors' contributions

MB and VD conceived the study and provided the research idea; VM, LBie < and MB performed clinical work and surveyed the patients; MB, OP and VB performed the analysis, MB, VB and OP drafted the article. All authors read and approved the final manuscript
